# Machine learning model to predict mortality in patients with skin and soft tissue infection in emergency department

**DOI:** 10.1186/s13049-025-01463-7

**Published:** 2025-09-24

**Authors:** Yu-Wei Chen, Kai-Hsiang Wu, Po-Han Wu, Cheng-Ting Hsiao, Chiao-Hsuan Hsieh, Wen-Chih Fann, Leng-Chieh Lin, Chia-Peng Chang

**Affiliations:** 1 Department of Emergency Medicine, Chiayi Chang Gung Memorial Hospital, No. 6, W. Sec., Jiapu Rd. Chiayi County, Puzih City, 613 Taiwan; 2 Department of Nursing, Chang Gung University of Science and Technology, Chiayi Campus, No.2, Sec. W., Jiapu Rd., Chiayi County, Puzi City, 613 Taiwan; 3 Shu-Zen Junior College of Medicine and Management, No. 452, Huanqiu Rd., Luzhu, Kaohsiung, 821 Taiwan; 4 Graduate Institute of Clinical Medical Sciences, College of Medicine, Chang Gung University, Taoyuan, 333 Taiwan

**Keywords:** Skin and soft tissue infection, Mortality, Artificial intelligence, Machine learning

## Abstract

**Background:**

Accurately predicting mortality in patients with skin and soft-tissue infections (SSTIs) remains challenging. Machine learning models offer rapid processing, algorithmic impartiality, and strong predictive accuracy, which may improve early risk stratification in the emergency department (ED).

**Methods:**

We retrospectively analyzed clinical data from 1,294 ED patients diagnosed with SSTIs between March 2015 and December 2020. Five machine learning algorithms—logistic regression (LR), k-nearest neighbours (KNN), support vector machine (SVM), random forest (RF), and Extreme Gradient Boosting (XGBoost)—were developed using 20 candidate variables, with model performance evaluated in independent runs. A simplified XGBoost model using only the six most influential predictors was also derived for bedside application.

**Results:**

Among the five models, XGBoost achieved the highest performance (AUC = 0.892, sensitivity = 86.9%, specificity = 93.4%). The streamlined six-variable XGBoost model further improved predictive metrics (AUC = 0.922, sensitivity = 88.5%, specificity = 95.4%), matching or slightly surpassing the full model while reducing data requirements.

**Conclusions:**

XGBoost outperformed LR, KNN, SVM, and RF in predicting SSTI mortality, offering both higher accuracy and operational efficiency. Its sequential tree-building, regularization, and robust handling of missing data enable superior discrimination in tabular clinical datasets. The simplified model, requiring only standard admission variables, provides a fast, cost-effective, and highly accurate tool for early identification of high-risk patients in the ED.

**Supplementary Information:**

The online version contains supplementary material available at 10.1186/s13049-025-01463-7.

## Introduction

Skin and soft-tissue infections (SSTIs) comprise a spectrum of disorders produced by microbial invasion of the skin, subcutaneous tissue, fascia, or muscle. Contemporary clinical guidelines divide SSTIs into uncomplicated vs. complicated infections; necrotizing soft-tissue infections (NSTIs) represent the most severe end of the continuum, often requiring emergency surgery and critical-care support [1]– [2]. SSTIs are among the most common infectious diagnoses in emergency departments and primary care. A U.S. population-based study of 48 million person-years reported a stable overall incidence of 48 cases per 1,000 person-years between 2005 and 2010, with 95% managed entirely as out-patients [3].

By contrast, NSTIs are rare but life-threatening, with an estimated annual incidence of 0.4 per 100,000 population in the United States [4].

Recent years have seen significant advances in applying machine learning and deep learning to clinical outcome prediction. Metaheuristic learning strategies, such as hybrid optimization algorithms, have proven effective for feature selection and tuning neural networks—for instance, applying a Greylag Goose Optimization (GGO) algorithm paired with Long-Short Term Memory (LSTM) yielded notably high classification accuracy in cardiovascular disease prediction [5–7]. In a different domain, multitask deep learning architectures demonstrated strong performance in jointly predicting mechanical ventilation needs and associated mortality using ICU data [8]– [9]. AI frameworks have been applied to empower glioma prognosis through transparent ML models, offering interpretative insights for clinical decision-making [10]. Similarly, deep neural networks combined with AI approaches have achieved high accuracy in detecting sickle cell disease, providing clinicians with interpretable predictions that can be integrated into diagnostic workflows [11]. In obstetric care, explainable AI-driven models have been developed for gestational diabetes mellitus prediction using clinical and laboratory markers, enabling early risk stratification and targeted interventions [12]. These recent works underscore the growing importance of integrating interpretability into ML models for clinical application, a principle that underpins the present study’s development of an explainable prediction model for mortality in patients with skin and soft tissue infections in the emergency department. These studies showcase the potential of combining model optimization and deep neural networks for critical care risk stratification .

Nevertheless, most prior work assesses broad critical illness outcomes or imaging-based diagnosis. There remains a lack of focused machine learning–based tools tailored to predicting mortality specifically in emergency department patients with SSTIs. Our study fills this gap by leveraging structured admission data to develop and evaluate diverse machine learning models and transforming the best performer into a streamlined, bedside-compatible tool for early mortality risk assessment in SSTI patients.

Integrating risk prediction, prompt multidisciplinary care, and timely interventions represents the key opportunity for future reductions in SSTI-related deaths. This study applied machine learning techniques to analyze ED data on patients with SSTIs and to build a mortality-prediction model. Early identification of high-risk SSTI patients in the ED could help curb the rising mortality trend among older, multimorbid populations.

## Materials and methods

### Patient selection

The Institutional Review Board of Chang Gung Memorial Hospital, Chiayi, Taiwan, approved this retrospective study. We retrospectively collected data on patients who had a discharge diagnosis of SSTI, identified according to the International Classification of Diseases, Ninth Revision, Clinical Modification (ICD-9-CM) codes 528.3 (cellulitis and abscess of oral soft tissues), 681.00–681.9 (cellulitis and abscess of the finger and toe), and 682.0–682.9 (other cellulitis and abscess), 728.86 (necrotizing fasciitis) and who was admitted via our ED from March 2015 to December 2020. Each patient’s medical record was reviewed for documentation of SSTI or a related diagnosis, clinical characteristics, final diagnosis, hospitalization duration, comorbidities and mortality. Patients aged ≤ 18 years, those found not to have had SSTI on chart review, and those having a discharge diagnosis of another infectious disease were excluded from the study.

### Data collection and analysis

All patients who visited our ED between March 2015 to December 2020, who had an discharge diagnosis with SSTI, and who did not meet the exclusion criteria were enrolled in our study. We reviewed the patients’ charts and recorded the following variables: age, sex, clinical condition, site of infection, laboratory data, comorbidities, hospital stay, blood culture result, and survival status on discharge. All blood tests were conducted within an hour of ED arrival.

Data analysis was performed with SPSS v 22.0. Categorical variables are reported as counts with corresponding percentages, whereas continuous data are summarised as medians and ranges. Group differences were examined with the χ² test for categorical variables. A two-sided *P* < 0.05 denoted statistical significance, and variables meeting this threshold were forwarded to model development.

To predict mortality, we built five machine-learning models—RF, LR, KNN, SVM, and XGBoost. LR, KNN and SVM required preprocessing: all input features were normalised to remove unit-scale effects while preserving their original distributions, and missing values were replaced with the normalised mean. The RF algorithm could accommodate raw inputs, so no additional preprocessing was applied.

For each algorithm, hyperparameter optimization was conducted using a grid search strategy combined with 5-fold cross-validation within the training cohort (80% of the dataset). Candidate parameter ranges were selected based on prior literature and preliminary trials. For LR, we tuned the regularization type (L1 vs. L2) and inverse regularization strength (C). For KNN, we varied the number of neighbours (k = 3–15) and distance metrics (Euclidean, Manhattan). For SVM, we optimized the kernel type (linear, radial basis function), regularization parameter (C), and kernel coefficient (γ). For RF, we tuned maximum tree depth (5–30), and minimum samples per split (2–10). For XGBoost, we adjusted the learning rate (0.01–0.3), maximum tree depth (3–10), subsample ratio (0.5–1.0), column sample by tree (0.5–1.0), and L1/L2 regularization weights. The optimal hyperparameter set for each algorithm was selected by maximizing the mean area under the ROC curve (AUC) across the 5 folds, and the final model was retrained on the entire training set before evaluation on the hold-out test set (20% of the dataset).

Model performance was evaluated using sensitivity, specificity, precision, F1 score, accuracy, Receiver Operating Characteristic Area Under the Curve (ROC-AUC), and Precision–Recall Area Under the Curve (PR-AUC). ROC-AUC was used to assess the overall discriminatory ability between survivors and non-survivors, while PR-AUC was included to better capture performance in the minority class (mortality) given the imbalanced outcome distribution. Sensitivity and specificity quantified the model’s ability to correctly identify true positives and true negatives, precision measured the proportion of predicted positive cases that were correct, and the F1 score provided a balanced measure of precision and recall. Accuracy was reported for completeness but interpreted cautiously due to the skewed class distribution.

## Results

### Patient characteristics

Of 1294 patients, 41 patients died. Therefore, the mortality rate was 3.2%. Compared with survivors, non-survivors were older and had higher rates of comorbidities such as diabetes, chronic kidney disease, and cirrhosis. They also presented with more pronounced systemic inflammation, renal impairment, and hemodynamic instability, and had longer hospital stays. Detailed comparisons of all variables, including percentages and *p*-values, are provided in Table 1. Collectively, these findings highlight that advanced age, metabolic and end-organ comorbidities, intense inflammatory response, renal impairment, and early hypotension are all strong correlates of mortality in SSTIs.

**Table 1:** Clinical characteristics of the mortality and survival groups in SSTI

Variable	Mortality group (*n* = 41)		Survival group (*n* = 1253)		*P* value
Age > = 65, No. (%)	31	(75.6%)	738	(58.9%)	< 0.001^*^
Sex, No. (%)					0.377
Male	23	(56.1%)	772	(61.6%)	
Female	18	(43.9%)	481	(38.4%)	
Diabetes mellitus	22	(53.7%)	426	(34.0%)	0.003^*^
CKD	20	(48.8%)	351	(28.0%)	< 0.001^*^
Liver cirrhosis	10	(24.4%)	152	(12.1%)	0.001^*^
Adrenal insufficiency	3	(7.3%)	61	(4.9%)	0.596
Malignancy	4	(9.8%)	150	(12.0%)	0.732
WBC count > 10 × 10^3^/µL	22	(53.7%)	476	(38.0%)	0.001^*^
WBC count < 4 × 10^3^/µL	3	(7.3%)	122	(9.7%)	0.179
Bandemia	18	(43.9%)	112	(8.9%)	< 0.001 ^*^
Hemoglobin < 10 mg/dL	13	(31.7%)	275	(21.9%)	0.458
Platelet counts < 10 × 10^4^ µL	10	(24.4%)	177	(14.1%)	0.02^*^
Serum glucose > 200 mg/dL	18	(43.9%)	363	(29.0%)	0.04^*^
Systolic BP < 90 mmHg	11	(26.8%)	97	(7.7%)	< 0.001 ^*^
CRP > 100 mg/L	18	(43.9%)	152	(12.1%)	< 0.001^*^
Sodium < 135 mEq/L	5	(12.2%)	225	(17.9%)	0.968
Creatinine > 1.6 mg/dL	15	(36.6%)	163	(13.0%)	0.02^*^
BT > = 38℃	14	(34.1%)	288	(23.0%)	0.137
BT < 35℃	4	(9.8%)	116	(9.3%)	0.742
Hospital stay (day)	13.4	(± 6.8)	9.3	(± 5.5)	0.04^*^

Values are presented as mean ± standard deviation or number (%)

*p < 0.05

*BP* blood pressure, *BT* body temperature, *CKD* chronic kidney disease, *WBC* white blood cell, *CRP* C-reactive protein

### Features derived from clinical parameters and comorbidity

Among those 20 features, 12 features were significantly different between mortality and survival patients. Those significant 12 features were throwed into the model: age > 9 = 65, diabetes mellitus; chronic kidney disease (CKD), liver cirrhosis, white blood cell (WBC) count > 10 × 10^3^/µL, bandemia, platelet counts < 10 × 10^4^ µL, serum glucose > 200 mg/dL, systolic blood pressure (BP) < 90 mmHg, C-reactive protein (CRP) > 100 mg/L, creatinine > 1.6 mg/dL, hospital stay.

### Diagnostic performances among different machine learning algorithms

**Fig. 1:**
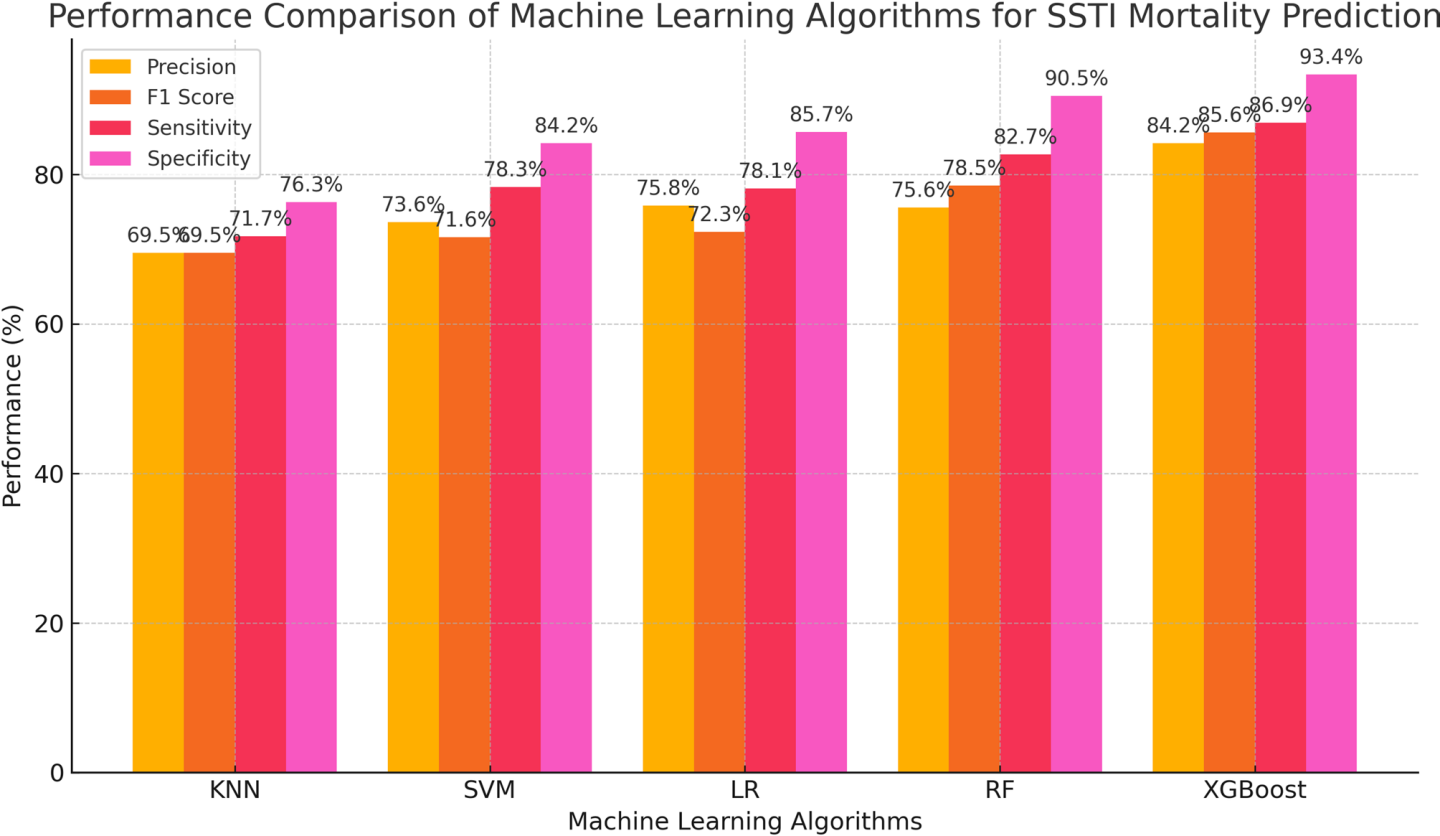
XGBoost emerged as the top-performing algorithm, yielding superior sensitivity, specificity, and overall accuracy compared with LR, KNN, RF, and SVM

**Fig. 2:**
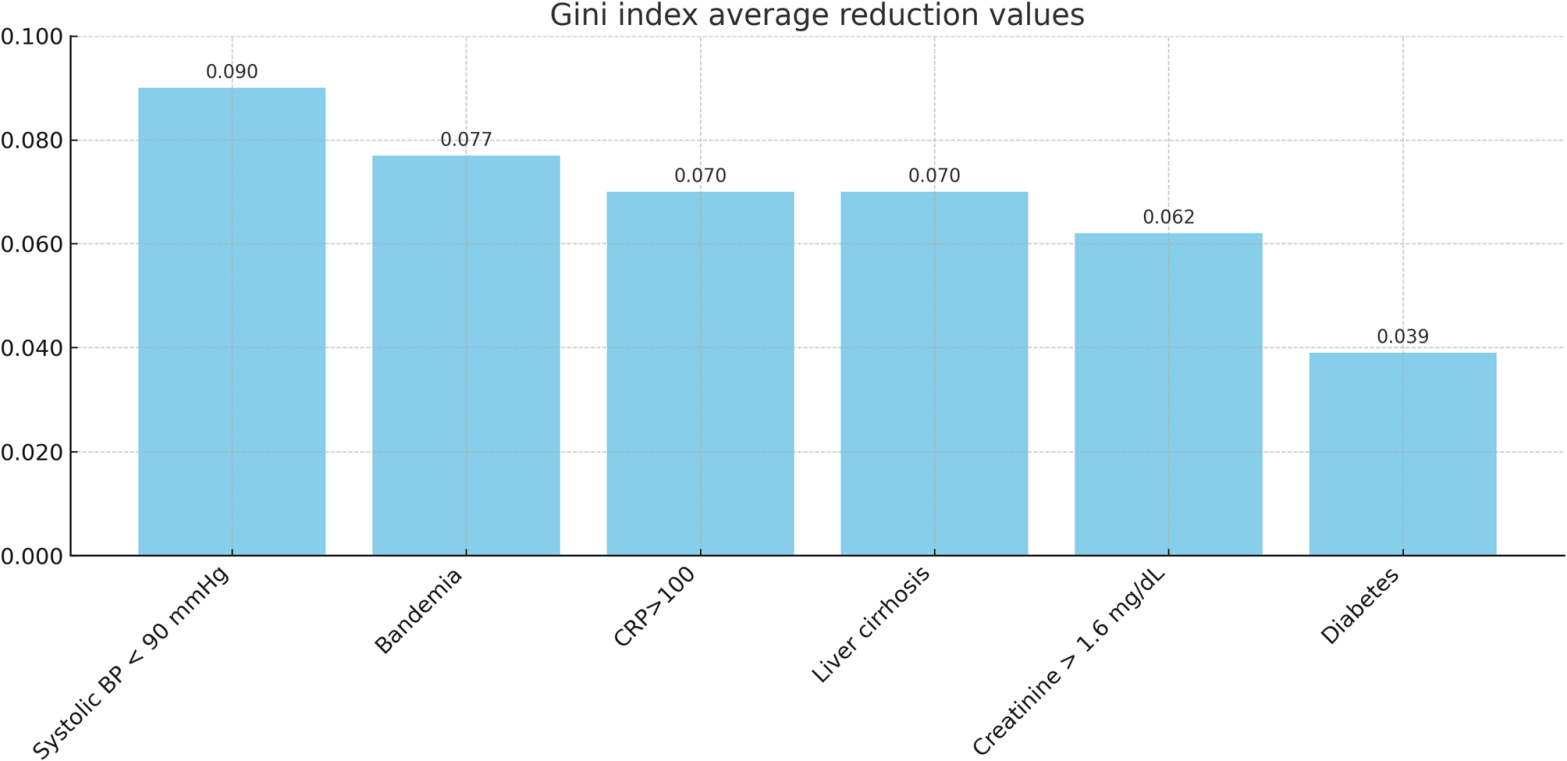
Feature importance ranking for the XGBoost model, based on average Gini index reduction values. The six most influential predictors—systolic blood pressure < 90 mmHg, bandemia, C-reactive protein > 100 mg/L, liver cirrhosis, creatinine > 1.6 mg/dL, and diabetes mellitus. BP: blood pressure; CRP: C-reactive protein

Table 2; Figs. 1 and 2 summarise how the five machine-learning methods fared in predicting mortality among SSTI patients. XGBoost emerged as the top-performing algorithm, yielding superior sensitivity, specificity, and overall accuracy compared with LR, KNN, RF, and SVM.

**Table 2:** Performance of machine learning algorithms to predict mortality of SSTI

	AUC	Sensitivity	Specificity	Precision	F1 score	Accuracy
KNN	0.785	71.7%	76.3%	69.5%	69.5	75.9%
SVM	0.823	78.3%	84.2%	73.6%	71.6	79.4%
LR	0.825	78.1%	85.7%	75.8%	72.3	80.1%
RF	0.879	82.7%	90.5%	75.6%	78.5	82.6%
XGBoost	0.892	86.9%	93.4%	84.2%	85.6	87.8%

AUC: Area under the ROC curve; KNN: k-nearest neighbor; LR: logistic regression; RF: random forest; SVM: support vector machine; XGBoost : eXtreme Gradient Boosting

To make the XGBoost tool more practical to use, we trimmed it to the six most influential variables, selected on the basis of their Gini index (see Fig. 2). These six predictors constitute the streamlined XGBoost model, whose performance metrics are presented in Table 3. Confusion matrices for the full 12-variable XGBoost model and the simplified 6-variable XGBoost model in the test set was shown in supplement material [S1]. The calibration plot of simplified XGBoost model was shown in supplement material [S2].

**Table 3:** Performance of new XGBoost model for predicting mortality of SSTI

	AUC	Sensitivity	Specificity	Precision	F1 score	Accuracy
XGBoost	0.922	88.5%	95.4%	86.8%	88.1	90.8%

AUC: Area under the ROC curve; XGBoost: eXtreme Gradient Boosting

Pairwise comparisons of ROC-AUC values between the best-performing model (simplified XGBoost) and each of the other four models using DeLong’s test. All *p*-values were adjusted using the Bonferroni correction for multiple comparisons. The results showed that the simplified XGBoost model had a significantly higher AUC compared to LR, KNN, and SVM (all adjusted *p* < 0.01), and a higher but non-significant difference compared to RF (adjusted *p* = 0.08). These findings support the superior discriminatory performance of the simplified XGBoost model in predicting SSTI mortality.

In addition to ROC-AUC, PR-AUC values for the five models were: LR 0.412, KNN 0.365, SVM 0.428, RF 0.501, and XGBoost 0.563. The simplified six-variable XGBoost model achieved the highest PR-AUC of 0.589, far above the baseline of 0.032 expected from random prediction given the class distribution. This indicates that the model maintained a strong balance between precision and recall in detecting mortality cases despite the highly imbalanced data.

## Discussion

Significant 12 features were age > = 65, diabetes mellitus; CKD, liver cirrhosis, WBC count > 10 × 10^3^/µL, bandemia, platelet counts < 10 × 10^4^ µL, serum glucose > 200 mg/dL, systolic BP < 90 mmHg, CRP > 100 mg/L, creatinine > 1.6 mg/dL, hospital stay. Severe SSTIs often progress to septic shock. During the initial response, perfusion is preferentially directed toward vital organs, including the heart and brain, at the expense of renal blood flow, causing early renal hypoperfusion. Subsequent fluid resuscitation and transfusions can intensify ischemia–reperfusion injury in the kidneys, producing a transient rise in serum creatinine. Deteriorating kidney function narrows both the dosing window and the range of antibiotics that can be prescribed, heightening the risk of sub-optimal antimicrobial therapy and mortality. In our prognostic model, hypotension and elevated serum creatinine were among the most influential predictors.

CRP, an acute-phase protein synthesised chiefly in the liver, remains a time-honoured marker of systemic infection. Markedly raised CRP concentrations often signal intense inflammation associated with deep soft-tissue involvement. Notably, in patients with decompensated cirrhosis, CRP has been shown to outperform procalcitonin and leukocyte count for detecting bacterial infection [13]. Among ED patients, CRP levels tend to rise in the setting of bacterial infection. Nevertheless, a single CRP result offers limited diagnostic precision; temporal changes in CRP are more reliable for anticipating mortality risk. Therefore, when the initial CRP value is markedly elevated, repeat measurements during the inpatient stay are recommended [14]– [15].

Our study examined the factors of clinical comorbidities are associated with mortality, which included diabetes mellitus and chronic kidney disease. High serum glucose levels are correlated with chronic kidney disease, diabetes mellitus, which often associated with chronic illness and poor nutrition status. An association between comorbidity and positivity rates of blood culture has been described before, especially for malignancy, immunodeficiency or diabetes mellitus [16]– [17]. Diabetes mellitus is a comorbidity with a high prevalence and relationship between diabetes mellitus and mortality has been described [18].

Multiple recent cohort studies demonstrate that both liver cirrhosis and thrombocytopenia markedly amplify infection-related mortality. Patients with cirrhosis who present to hospital already harbouring an infection face more than double the short-term risk of death or hospice referral (22.1% vs. 8.0%; adjusted risk ratio 1.75) compared with cirrhotics admitted for non-infectious reasons. When cirrhosis coexists with necrotizing fasciitis, in-patient lethality rises from 3 to 9.5%, an almost four-fold increase (adjusted OR 3.78), and episodes of septic shock are likewise more common [19]– [20]. Thrombocytopenia, whether pre-existing or infection-induced, has a similar prognostic impact. In a prospective study of ≥ 65-year-old medical in-patients, non-resolving thrombocytopenia drove in-hospital mortality from 12.7 to 24.5% [21]. Consistent with this, an admission platelet count emerged as the single most powerful predictor of death in a 389-case series of severe SSTIs [22]. These data underscore that compromised hepatic reserve and low platelet counts signal a host unable to contain infection-driven systemic injury, translating into dramatically worse outcomes once SSTIs or other severe infections take hold. Early recognition of these comorbidities should therefore prompt more aggressive monitoring and tailored antimicrobial and supportive strategies.

Recent studies indicate that an elevated proportion of bandemia is a straightforward yet powerful predictor of infection-related mortality. In a five-year retrospective cohort of 138 patients with necrotizing soft-tissue infection, an admission band count exceeding 25% increased the risk of in-hospital death eight-fold [23]. Similar findings emerge in broader ED cohorts: a prospective study across two community hospitals showed that moderate bandemia (11–19%) and severe bandemia (≥ 20%) were independently associated with 3.2-fold and 4.7-fold higher in-hospital mortality, respectively, even when total leukocyte counts were normal, and they also correlated with a greater likelihood of Gram-negative bacteraemia [24].

Artificial intelligence has rapidly reshaped many sectors in recent years, and its foothold in medicine continues to expand. In diagnostics, machine learning methods can ingest large, heterogeneous datasets and distill them into highly predictive models. A variety of algorithms are already deployed in clinical research and practice. LR is a long-standing statistical tool that outputs a probability between 0 and 1; values ≥ 0.5 are typically interpreted as a positive class. Its transparency and ease of implementation make it a staple for clinical-risk calculators. KNN assigns an unlabelled example to the class most common among its *k* closest training neighbours, effectively performing a “majority vote” in feature space. SVM construct an optimal hyper-plane that maximizes the margin between different classes, allowing robust binary and multi-class separation. RF aggregate the predictions of many decision trees. Each tree learns a set of IF–THEN rules; the ensemble’s final classification is derived from the collective “votes” of those trees. Ensembling mitigates the over-fitting that can plague deep, single trees and generally yields more stable performance.

XGBoost has emerged as a front-runner in tabular medical data. Unlike RF, which average many uncorrelated trees grown in parallel, XGBoost builds trees sequentially, with each new tree focusing on the residual errors of the preceding ensemble. Regularisation terms, shrinkage, and colxumn subsampling further control over-fitting, while built-in handling of missing values streamlines preprocessing. These design choices often translate into faster training and superior accuracy, making XGBoost a popular “go-to” algorithm for contemporary diagnostic-prediction tasks [25].

While ROC-AUC remains a widely used performance metric, it may present overly optimistic results in the presence of highly imbalanced outcomes. In our dataset, where mortality accounted for only 3.2% of cases, PR-AUC provides a more informative measure of model utility for the minority class. The simplified XGBoost model achieved a PR-AUC of 0.589, substantially exceeding the baseline PR-AUC of 0.032 that would be expected from random guessing. Previous studies have evaluated the performance of the National Early Warning Score (NEWS) and the quick Sequential Organ Failure Assessment (qSOFA) in predicting in-hospital mortality among patients with suspected infection in the emergency department. Reported AUC values for NEWS generally range between 0.84 ~ 0.86 [26], while qSOFA typically achieves AUC values between 0.81 [27]. In our cohort of SSTI patients, the simplified XGBoost model achieved a AUC of 0.922, substantially higher than the ranges reported for these generic sepsis screening tools. This difference likely reflects the fact that NEWS and qSOFA rely predominantly on vital signs and mental status, whereas our model incorporates infection-relevant laboratory markers and comorbidities—such as CRP > 100 mg/L, creatinine > 1.6 mg/dL, and liver cirrhosis—that are particularly informative for SSTI prognosis. As a result, our model provides a more SSTI-specific risk stratification, enabling more precise identification of high-risk patients at the point of ED presentation.

In our research, we created predictive models for SSTI mortality utilizing five machine learning algorithms. We then compared predictive accuracy of these models to identify the most effective one for predicting death. We incorporated 12 features that exhibited statistical variances between mortality and survival groups into the model. These included blood pressures, blood metrics, and comorbidities. Given that all these measures are extensively utilized in clinical settings, they can be quickly and easily procured without requiring any specialized apparatus. As a result, the models illustrated in this paper hold broad applicability.

The findings indicate that among the five algorithms, XGBoost displays the most superior predictive performance with a sensitivity, specificity, accuracy, and AUC of 86.9%, 93.4%, 87.8%, and 0.892 respectively. The diagnostic performances of LR, SVM, RF were found to be alike. XGBoost is often preferred over other machine learning approaches in clinical prediction tasks. Each study directly compared XGBoost with at least two traditional algorithms and found it delivered the best discrimination [28–30].

To make the tool more practical at the bedside, we trimmed the original XGBoost model from 12 variables down to the six predictors with the highest importance scores. Despite this reduction, the simplified model achieved diagnostic performance comparable to the full version. Relying on fewer inputs can decrease laboratory expenditures and streamline decision-making for clinicians. Several caveats should be acknowledged. All data came from a single institution, and the sample size was relatively modest. Future work should therefore test the model prospectively across multiple centres with a larger cohort, which would improve generalisability and may further refine mortality prediction in SSTI.

This study has several limitations. First, it was conducted at a single tertiary care center in Taiwan, which may limit the generalizability of the model to other institutions with different patient populations, clinical practices, and laboratory protocols. External validation in multi-center, geographically diverse cohorts is necessary to confirm the model’s robustness. Second, the retrospective design carries inherent risks of selection bias and incomplete documentation, and causality cannot be inferred from the observed associations. Third, although we took care to prevent information leakage by splitting the dataset into training and testing sets prior to model fitting and by performing all preprocessing steps independently within the training set, we cannot fully exclude the possibility of indirect leakage (e.g., through shared normalization parameters) influencing the results. Future prospective implementation should incorporate strict data separation protocols. Fourth, the low mortality rate (3.2%) introduced class imbalance, which we addressed using stratified sampling to preserve event proportion between datasets; however, model performance in settings with different prevalence may vary. Prospective trials should also assess how the model performs in real-time clinical decision-making, particularly in rare-event prediction scenarios. Future work should focus on prospective validation and integrating the model into electronic health record (EHR) systems could enable automated, real-time mortality risk calculation upon patient arrival in the emergency department, reducing cognitive load for clinicians.

## Conclusion

Leveraging machine learning tools to predict mortality in SSTIs can sharpen clinical vigilance and accelerate lifesaving therapy. In our comparisons, XGBoost consistently surpassed LR, KNN, RF and SVM. A streamlined XGBoost model that draws only on standard admission data could deliver a fast, low-cost bedside decision aid, ultimately helping clinicians target high-risk patients earlier and more effectively.

## Supplementary Information

Below is the link to the electronic supplementary material.

Supplementary Material 1

Supplementary Material 2

## Data Availability

No datasets were generated or analysed during the current study.

## Abbreviations

BP: Blood pressure
, BT: Body temperature
CKD: Chronic kidney disease
CRP: C-reactive protein
ED: Emergency department
GGO: Greylag goose optimization
KNN: K-nearest neighbor
LR: Logistic regression
LSTM: Long-short term memory
NEWS: National early warning score
NSTIs: Necrotizing soft-tissue infections
PR-AUC: Precision–recall area under the curve
qSOFA: Quick sequential organ failure assessment
RF: Random forest
ROC-AUC: Receiver operating characteristic area under the curve
SD: Standard deviation
SSTI: Skin and soft tissue infection
SVM: Support vector machine
WBC: White blood cell
XGBoost: Extreme gradient boosting
