# Supplementary figures and images for: Machine learning model to predict mortality in patients with skin and soft tissue infection in emergency department
Source: Scand J Trauma Resusc Emerg Med. 2025 Sep 24;33:148. doi: 10.1186/s13049-025-01463-7 (PMC12462322; doi:10.1186/s13049-025-01463-7)

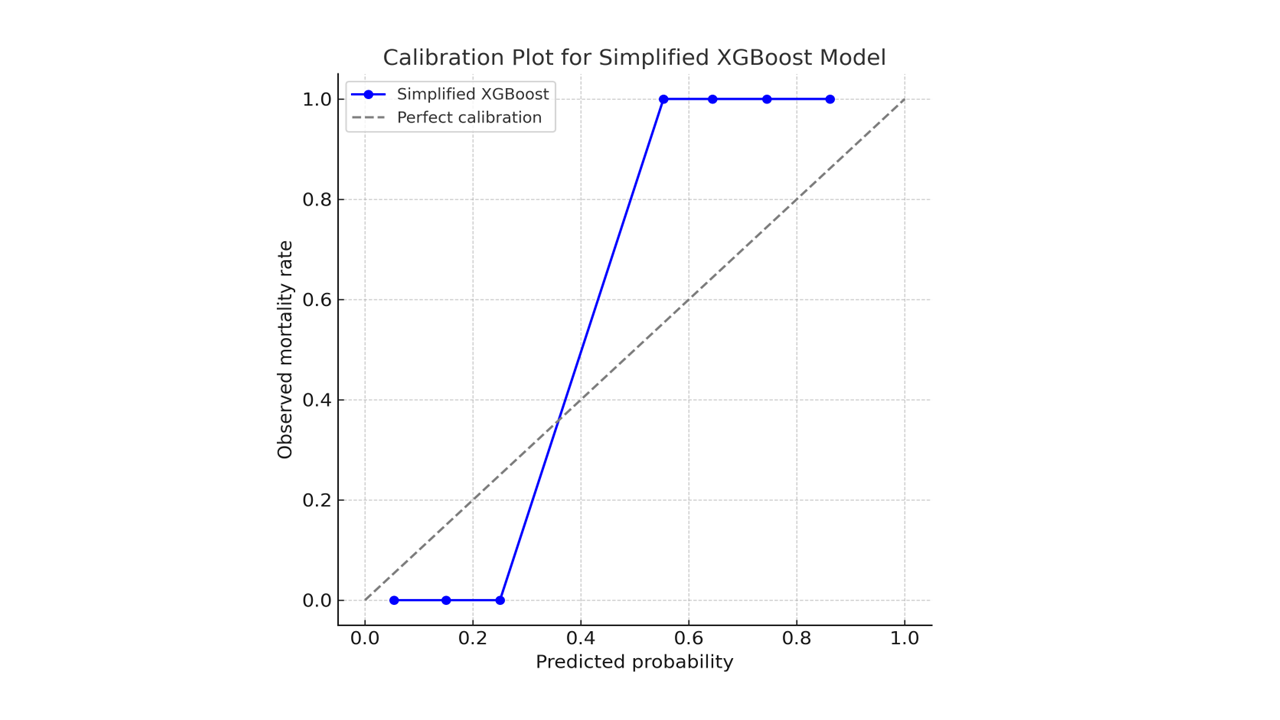

Supplement: Supplementary file 1 — Supplementary Material 1 [file 13049_2025_1463_MOESM1_ESM.tif]

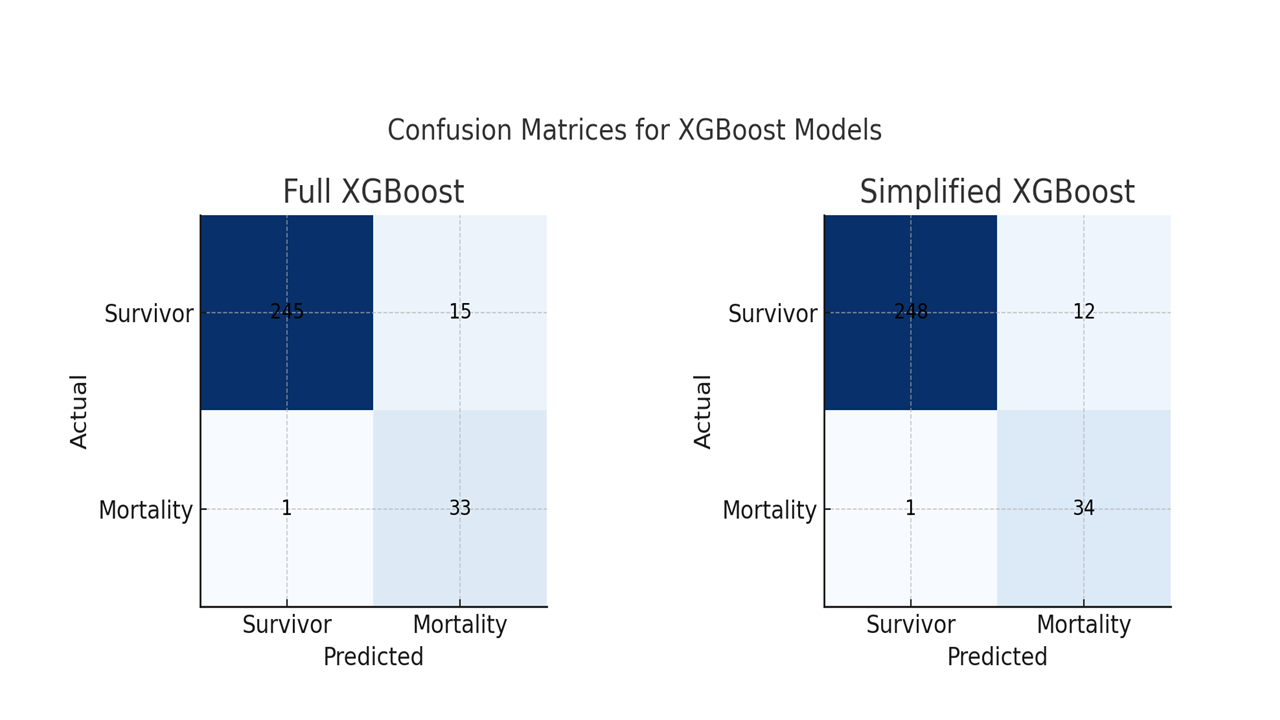

Supplement: Supplementary file 2 — Supplementary Material 2 [file 13049_2025_1463_MOESM2_ESM.tif]
